# Evaluating the Feasibility of a Digital Therapeutic Program for Patients With Cancer During Active Treatment: Pre-Post Interventional Study

**DOI:** 10.2196/39764

**Published:** 2022-10-13

**Authors:** G Haukur Gudmundsson, Judit Mészáros, Ágústa E Björnsdóttir, María L Ámundadóttir, Gudrun E Thorvardardottir, Erna Magnusdottir, Halla Helgadottir, Saemundur Oddsson

**Affiliations:** 1 Medical and Research Department Sidekick Health Kopavogur Iceland; 2 Medical and Research Department Sidekick Health Berlin Germany; 3 Physical rehabilitation Ljosid Cancer Rehabilitation Centre Reykjavík Iceland; 4 Management Ljosid Cancer Rehabilitation Centre Reykjavík Iceland

**Keywords:** cancer, lifestyle, quality of life, mobile app, digital therapeutics, self-management, physical activity, mobile phone

## Abstract

**Background:**

Increasing evidence shows that lifestyle interventions can improve the symptoms, quality of life (QoL), and even overall survival of patients with cancer. Digital therapeutics (DTx) can help implement behavioral modifications and empower patients through education, lifestyle support, and remote symptom monitoring.

**Objective:**

We aimed to test the feasibility of a DTx program for patients with cancer, as measured by engagement, retention, and acceptability. In addition, we explored the effects of the program on cancer-related QoL.

**Methods:**

We conducted a 4-week single-arm trial in Iceland, where DTx was delivered through a smartphone app. The intervention consisted of patient education about mindfulness, sleep, stress, and nutrition; lifestyle coaching; and the completion of daily missions for tracking physical activity and exercise, reporting patient-reported outcomes (PROs), practicing mindfulness, and logging healthy food intake. Information on program engagement and retention, step goal attainment, as well as PROs were collected throughout the study. QoL was measured using the European Organization for Research and Treatment of Cancer Quality of Life Questionnaire C30 at baseline and follow-up.

**Results:**

In total, 30 patients with cancer undergoing active therapy were enrolled, and 29 registered in the app (23 female, 18 with breast cancer; mean age 52.6, SD 11.5 years). Overall, 97% (28/29) of participants were active in 3 of the 4 weeks and completed the pre- and postprogram questionnaires. The weekly active days (median) were 6.8 (IQR 5.8-6.8), and 72% (21/29) of participants were active at least 5 days a week. Users interacted with the app on average 7.7 (SD 1.9) times per day. On week 1, all 29 participants used the step counter and logged an average of 20,306 steps; 21 (72%) participants reached their step goals of at least 3000 steps per day. On week 4, of the 28 active users, 27 (96%) were still logging their steps, with 19 (68%) reaching their step goals. Of the 28 participants who completed the satisfaction questionnaire, 25 (89%) were likely to recommend the program, 23 (82%) said the program helped them deal with the disease, and 24 (86%) said it helped them remember their medication. QoL assessment showed that the average global health status, functioning, and symptom burden remained stable from baseline to follow-up. In all, 50% (14/28) of participants reported less pain, and the average pain score decreased from 31 (SD 20.1) to 22.6 (SD 23.2; *P*=.16). There was no significant change in PROs on the quality of sleep, energy, and stress levels from the first to the last week.

**Conclusions:**

The high retention, engagement, and acceptability found in this study demonstrate that multidisciplinary DTx is feasible for patients with cancer. A longer, full-scale randomized controlled trial is currently being planned to evaluate the efficacy of the intervention.

## Introduction

According to the latest statistics, the global prevalence of all types of cancers is projected to increase by nearly 50% in the next 20 years, with female breast cancer being the most prevalent in 2020 [1]. In Iceland, approximately 1700 people received a new cancer diagnosis in 2018, but due to improved awareness, early detection, and treatment options, survival rates have been increasing, and by 2018, there were >15,000 people living with a previously diagnosed cancer [2,3]. Thus, there is now an increased population of people living a long, productive life with a history of cancer. Nevertheless, the side effects of current treatments, as well as the stress associated with the diagnosis and fear of disease recurrence, pose a serious burden on patients’ mental and physical well-being [4,5].

Research over the last 2 decades has shown that lifestyle modifications can effectively improve the quality of life (QoL) of patients with cancer. Mindfulness exercises, muscle relaxation, and cognitive behavioral therapy can help patients cope with stress [6]. Traditionally, patients were advised to rest while undergoing cancer treatment, but guidelines now recommend avoiding sedentary behaviors and doing regular aerobic and resistance training [7]. Increasing evidence suggests that regular physical activity can help combat disease-related physical and psychological symptoms and improve QoL [7]. Research has shown that more walking during recovery can reduce the chance of readmission after cancer surgery and engaging in home-based exercise programs can aid people in increasing their physical fitness [8,9]. In addition, healthy dietary habits are equally important to maintain for patients with cancer and cancer survivors [10]. However, lifestyle and related behavioral modifications are often hard to achieve, and there is a need for a structured implementation of lifestyle support for patients.

The advent of digital technology and the wide reach of smartphones provide a potential avenue for motivating and delivering structured lifestyle programs for patients. Several digital intervention programs have been developed for patients with cancer and cancer survivors to provide psychological support and help manage symptoms [11]. Studies integrating electronic patient-reported outcomes (PROs) and advice on symptom management in care found reduced fatigue and increased QoL and overall survival [12-14]. In addition, collecting electronic PROs can foster more efficient patient–health care provider interaction and thus reduce the need for consultations [15]. Studies on digital therapeutics (DTx) for patients with cancer have been largely focused on providing and evaluating psychosocial interventions. These studies found that electronically delivered cognitive behavioral therapy increases the ability to relax and decreases anxiety in men with prostate cancer [16], combining it with exercise and patient education can increase QoL, reduce fatigue, and improve dietary habits [17,18]. Furthermore, a study found that an internet-based exercise program in itself can improve cognitive abilities in patients with breast cancer for 6 months after the intervention [19]. Thus, existing evidence points toward the benefits of a holistic digital therapeutic that combines multiple aspects of patient support during cancer treatment (psychological support, education, exercise, and nutrition).

The primary aim of this study was to assess the feasibility of a holistic DTx program to improve the lifestyle and health-related QoL of patients in active anticancer therapy. This was measured in terms of user engagement, retention, acceptability, and step goal attainment. An additional objective of the study was to gather preliminary indications of the program’s efficacy through secondary endpoint measures. The results of this feasibility trial will be used to inform a future definitive randomized controlled trial (RCT).

## Methods

### Study Design and Recruitment

We conducted a 4-week single-arm trial from August to November 2021 at the Ljósið cancer rehabilitation clinic in Iceland. Patients were invited to participate in the study, which was promoted as a support program aimed at improving QoL for patients with cancer, via emails and educational lectures at the clinic, and they were recruited after voluntarily reaching out. Inclusion criteria were (1) diagnosed with cancer and receiving anticancer treatment at the National University Hospital of Iceland (chemotherapy, radiation therapy, or other nonhormonal cancer medication) at the start of participation; (2) aged ≥18 years; (3) speaks Icelandic; (4) has the capacity to give informed consent; and (5) owns and knows how to operate a smartphone.

### Ethical Considerations

All participants provided informed consent before enrolling in the study. The protocol was approved by the National Bioethics Committee (institutional review board registration number VSN-21-102). This study was conducted in accordance with the ethical principles of the Declaration of Helsinki 2008.

### Procedures

After signing informed consent forms, participants completed a preintervention QoL questionnaire on the web, and exercise physiologists or physical therapists at the cancer clinic collected information about baseline physical measurements, physical fitness and body composition. Participants were also instructed to download the Sidekick smartphone app and received an access code to the program. During the intervention, data on participants’ retention, engagement, and self-reported in-app activity were collected through the app. QoL, fitness, and body composition measurements were repeated after the intervention, and participants’ feedback on the program was collected through a web-based satisfaction questionnaire.

### Intervention

The program was delivered through the Sidekick app, which was created by a group of data scientists, designers, gamification experts, behavioral scientists, psychologists, medical doctors, and other health care professionals and uses the principles of behavioral economics combined with gamification elements to achieve behavioral modifications for the primary and secondary prevention of lifestyle-related chronic diseases [20]. Gamification elements in the app are intended to motivate and engage users and examples include the collection of digital water drops—which are converted into donations for charity—when completing any task in the app; a leveling system and progress bar based on the water drops collected; and a checklist for completion of daily tasks. In addition, the visual appearance, animations, and sounds within the app were designed to be enjoyable, pleasing, and mimic games.

**Figure 1:**
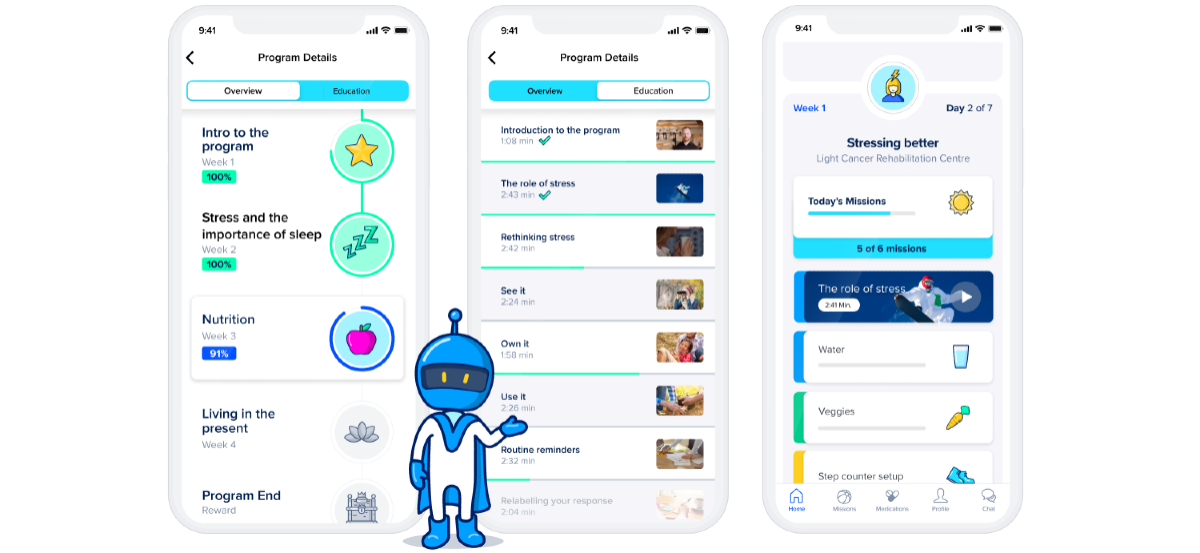
The Sidekick app interface. Program overview (left): the program was composed of 4 weekly modules with introduction on week 1, followed by modules on stress and sleep, nutrition, and mindfulness in the following weeks. Users could access educational videos in each topic (middle) as part of their daily missions. The missions included reaching step goals and logging food and water intake (right).

The intervention was designed to provide patients with cancer with tools to better deal with side effects during cancer treatment and to improve their overall QoL, with the main focus on stress management and improving sleeping habits. A general program overview is provided in Table 1 and Figure 1, and overview of the educational content is provided in the Multimedia Appendix 1. The intervention was designed with inputs from oncologists and was composed of assigned daily missions for educational materials relating to each weekly theme (stress, sleep, nutrition, and mindfulness from week 1 to 4), step counter, food logging, meditation, and PRO surveys. Missions were defined as any assignment available in the app, and users received on average 5 of these a day. Users could also proactively explore the app for other content that was not assigned (eg, exercises and medication reminders). An additional feature was remote patient monitoring in which an exercise physiologist with experience in cancer rehabilitation provided lifestyle coaching through weekly motivational messages and general support with the program.

**Table 1:** Presentation of main program missions and expected outcomes.

Mission type	Content or action	Outcome
Education	Videos and other content every day	Increased knowledge of tools for building healthy habits
Food logger	Log vegetables and water consumed every day	Healthier eating habits and better nutrition
Step counter	Log number of steps every day	Increased awareness and motivation for physical activity
Guided meditation	Meditate 3 times per week	Improved mental health and stress management
Patient-reported outcomes	Indicate energy levels, stress levels, and quality of sleep 3 times per week	Increased self-awareness

### Primary Outcomes

Retention and engagement were assessed using data on user-reported interactions by completing missions on the Sidekick app. Treatment completion was defined as being active in-app at least 3 of 4 weeks (or 75% of the program duration) and completing the prestudy and poststudy QoL questionnaires. Although previous studies found that attrition rates can be as high as 50% [21-23], we based this more ambitious target on previous engagement and completion rates found on our platform. Program retention was defined as the number of users who returned to the app during the last week of the study, and highly engaged users were those who were active in-app for at least five days a week. We focused on 2 measures of engagement: the number of days the participant was active in the app and the average number of mission interactions during active days.

### Secondary Outcomes

Participants’ health-related QoL was assessed using the European Organization for Research and Treatment of Cancer Quality of Life Questionnaire C30 (QLQ-C30). QLQ-C30 is a clinically validated and well-established questionnaire composed of 30 questions that measure QoL across 3 domains: functional scales (physical, role, emotional, cognitive, and social), symptom scales (fatigue, nausea or vomiting, pain, dyspnea, insomnia, appetite loss, constipation, diarrhea, and financial difficulties), and global health status [24].

Aerobic fitness was measured using the Åstrand submaximal test on a cycle ergometer (Monark) [25]. In this test, the participants were instructed to cycle for 6 minutes with a pedal frequency of 50 to 70 rpm and reach approximately 85% of the maximum heart rate based on age. Their maximum oxygen uptake (VO_2max_; mL/kg/min) was estimated based on the heart rate, workload (in watt), age, sex, body weight, and heart rate at the end of the test.

Body composition was measured using the InBody 770 bioelectrical impedance analyzer [26,27]. The participants were instructed to stand on the device platform with bare feet, stand upright, and hold on to the handles. Built-in scales and electrodes in the device measure body composition values, such as weight, fat mass, and fat-free mass (lean mass). Participants were instructed not to consume food at least two hours before their appointment, refrain from physical exercise earlier that day, and avoid putting creams or lotions on their bodies.

Step counts were automatically measured by the app based on data from the built-in accelerometer of the smartphone. However, at the end of each day, the steps had to be manually registered by the user by clicking *claim steps* in the app. The minimum step goal was set at 15,000 per week or 3000 at least 5 days per week.

PROs on quality of sleep, stress, and energy levels were measured using the app 3 times a week. Users received these as part of their daily missions on 3 random days of the week and were composed of a prompt (“Please indicate last night’s quality of your sleep/today’s energy level/today’s stress level”) and a 10-point visual analog sliding scale, where 10 represents the highest sleep quality, energy levels, or stress levels. Users could rate these at any time of the day but received no additional reminders for them.

### Data Analysis

Characteristics are presented as mean and SD with the corresponding number and percentage of participants. User engagement information is presented as medians and IQR for weekly active days and total active days (active days out of 28 days), as these were nonparametric variables. Active days were defined as days when the user logged at least one mission. The average number of daily mission interactions is the number of events a user completes per mission; it was calculated as total mission interactions divided by total active days and is presented as mean and SD. Step goal attainment is shown as the number and percentage of users who used a step counter in the first and last weeks, along with weekly step counts as mean and SD, and step goal attainment is shown as the percentage of users who used the step counter.

Scores from QLQ-C30 were calculated according to instructions in the scoring manual (open source) [24]. To calculate the score for each subscale (global health status, physical functioning, role functioning, fatigue, pain, etc), we averaged the raw scores given to each of the questions contributing to that subscale. Most questions were scored on a scale of 1 (not at all) to 4 (very much), except for the questions contributing to global health status, which could be scored from 1 to 7. This raw score was then linearly transformed to 0 to 100 so that all subscales had the same range of values. For functional scales and global health status, a higher number represents a higher level of functioning and higher QoL; conversely, a higher number means a higher symptom burden for symptom scales. The number and percentage of participants whose scores increased, decreased, or did not change after the study was calculated along with the scores.

PROs on sleep, stress, and energy levels were compared at the beginning and end of the study using 2-tailed paired *t* tests. The normalized change was calculated as the change in the weekly average score divided by the maximum possible gain to be able to compare rating scales in different directions (evaluating increased energy levels and quality of sleep but decreased stress levels). The correlation between preprogram QLQ-C30 scores and the in-app PROs during the first week was calculated using Spearman’s rank correlations.

## Results

### Baseline Characteristics

**Figure 2:**
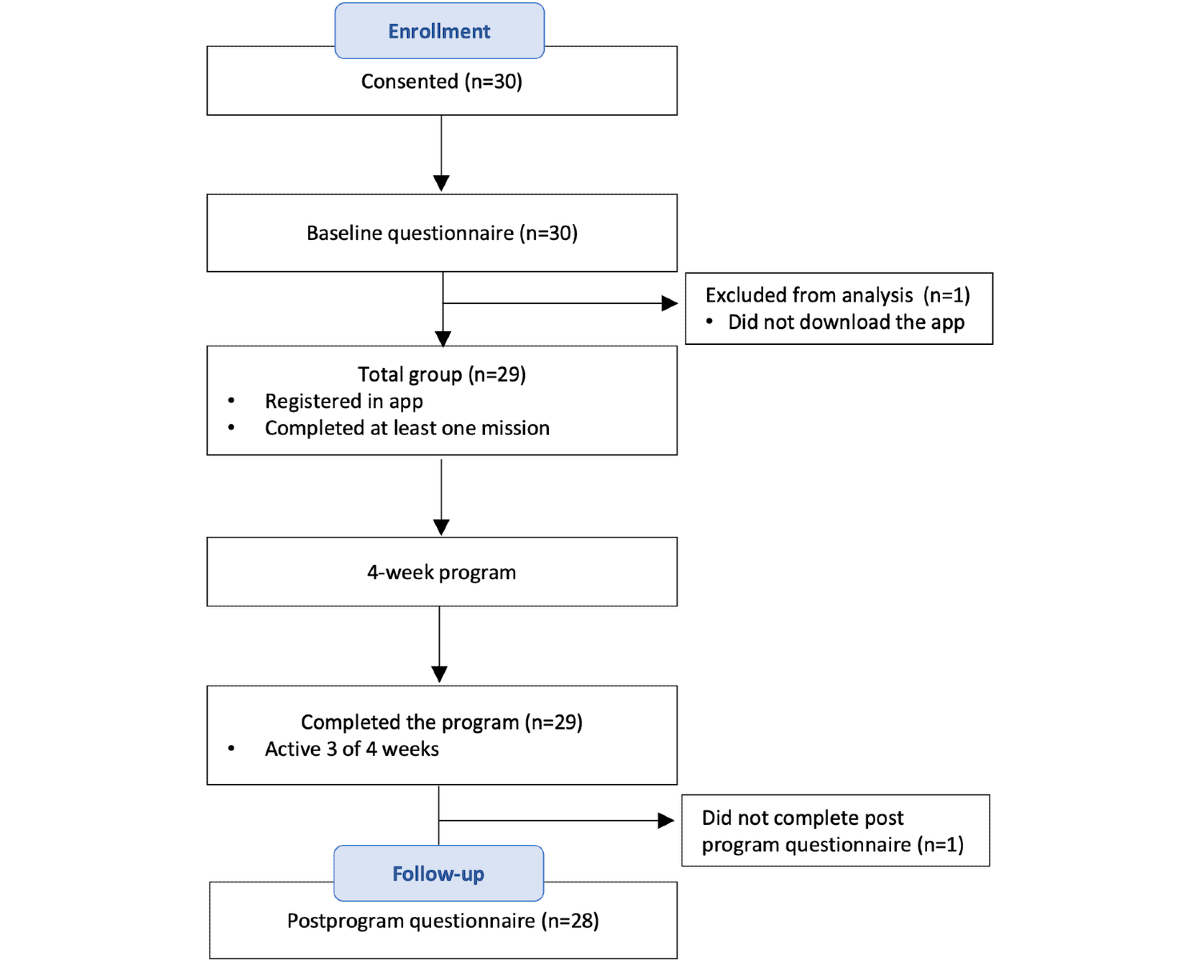
Participant flow through the study.

In total, 30 patients with cancer were initially enrolled in the program; 1 (3%) did not download the app and was therefore excluded from further analysis, and 29 (97%) completed the program with in-app activity in 3 of 4 weeks (Figure 2). Of the 29 participants, 1 (3%) was inactive in the last week but completed the follow-up questionnaire, and 1 (3%) participant who engaged with the program every week did not complete the postprogram questionnaire.

The baseline characteristics and measurements are shown in Table 2. Most participants were female (23/29, 79%); the average age was 52.6 (SD 11.5) years, and 62% (18/29) had breast cancer, while 38% (11/29) had other types of cancer. As there were 2 apparent patient groups with respect to cancer type (breast cancer and other cancer), we compared the baseline measurements of the 2 groups and found no statistically significant differences; thus, we present our results for the total patient population. A total of 28% (8/29) of participants had stage IV metastatic cancer, and 72% (21/29) had stage I to III cancer; all participants were receiving cancer treatment, either chemotherapy or radiation therapy, at the start of the study. The sample population was slightly obese, with an average BMI of 30 (SD 5.8) kg/m^2^, and most participants (23/29, 79%) were in the overweight or obese BMI ranges. As expected, the mean percentage of body fat was higher in females (41.2%) than in males (26.5%), and VO_2max_ was 27.7 mL/kg/min and 31.2 mL/kg/min in females and males, respectively. These measurements were repeated at the end of the study, but only small nonsignificant changes were observed (data not shown). Of the 29 participants, 28 (97%) completed the full program and all postprogram questionnaires.

**Table 2:** Participants’ baseline characteristics (N=29).

Characteristics			Value
Age (years), mean (SD)			52.6 (11.5)
Female, n (%)			23 (79)
**Cancer type and stage, n (%)**			
	Breast cancer		18 (62)
	Other cancer		11 (38)
	Stage I-III		21 (72)
	Stage IV (metastasis)		8 (28)
**Current therapy, n (%)**			
	Chemotherapy		26 (90)
	Radiation therapy		2 (7)
	Both		1 (3)
**BMI categories (kg/m^2^), n (%)**			
	Normal weight (18.5-24.9)		5 (17)
	Overweight (25.0-29.9)		9 (31)
	Obese (≥30.0)		14 (52)
**Body composition, mean (SD)**			
	Height (cm)		168.3 (7.5)
	Weight (kg)		85.0 (17.6)
	BMI (kg/m^2^)		30.0 (5.8)
	**Percentage body fat (%)**		
		Female^a^	41.2 (7.6)
		Male^b^	26.5 (8.6)
	**Fat mass (kg)**		
		Female^a^	32.6 (11.8)
		Male^b^	25.4 (17.5)
	**Lean mass (kg)**		
		Female^a^	48.1 (5.5)
		Male^b^	63.1 (9.3)
**Maximal aerobic capacity (VO_2max_^c^, mL/kg/min), mean (SD)**			
	Female^d^		27.7 (8.1)
	Male^e^		31.2 (11.6)

^a^Data available for 23 participants.

^b^Data available for 5 participants.

^c^VO_2max_: maximum oxygen uptake.

^d^Data available for 15 participants.

^e^Data available for 6 participants.

### Primary Outcomes

#### Program Feasibility

Engagement metrics are presented in Table 3. The median number of active days per week was 6.8 (IQR 5.8-6.8), while the total active days out of the maximum of 28 days of the program was 27 (IQR 23-27). Users interacted with missions on average 7.7 times a day, and the number of highly engaged users was 72% (21/29), of whom 57% (12/21) were patients with breast cancer and 43% (9/21) had other types of cancer. A total of 97% (28/29) participants continued using the app until week 4 (4-week retention), with only 1 with breast cancer being inactive in the last week.

Data on step counter use show that all 29 participants used this function in the first week, with 72% (21/29) achieving the set target. The number of users logging their steps remained high in the last week (27/28, 96% of active users), with 68% (19/28) reaching the target goal (Table 4). The average weekly step count increased by over 3000, from 21,307 in the first week to 24,449 in the last week.

**Table 3:** Engagement metrics (N=29).

Metric	Value
Completion rate^a^, n (%)	28 (97)
Weekly active days, median (IQR)	6.8 (5.8-6.8)
Total active days, median (IQR)	27.0 (23.0-27.0)
Daily mission interactions, mean (SD)	7.7 (1.9)
Highly engaged users^b^, n (%)	21 (72)
4-week retention, n (%)	28 (97)

^a^Completed 75% of the program and all preprogram and postprogram questionnaires.

^b^Users who were active in the app at least five days a week.

**Table 4:** In-app measured step counts and goal attainment (N=29).

Metric	Week 1	Week 4
Used step counter, n (%)	29 (100)	27 (96)
Attained step goal, n (%)	21 (72)	19 (68)
Weekly step counts, mean (SD)	21,306 (11,411)	24,449 (17,445)

#### Program Acceptability

Overall, 28 participants completed the postintervention satisfaction survey. These results showed that program acceptability was high, with 89% (25/28) of participants likely to recommend the program to others and 93% (26/28) who found the Sidekick app user friendly. Regarding program content, of the 28 participants, 26 (93%) found the educational content helpful, 23 (82%) said they felt better equipped to deal with their illness after participating in the program, and 24 (86%) said the app helped them remember to take their medication. With regard to the lifestyle coaching feature, 93% (26/28) of the participants said they found the weekly messages from the coach useful, but only 54% (15/28) somewhat agreed with the statement that they would have liked more feedback from the coach. Overall, 86% (24/28) of participants agreed that the program had positive effects on their lives and well-being.

### Secondary Outcomes

#### Quality of Life Questionnaire C30

The results of the self-reported QoL questionnaire are shown in Table 5. One individual did not complete the follow-up questionnaire; thus, only 28 completed questionnaires were analyzed. We found no significant change in the mean scores from inclusion to follow-up for any of the functional or symptom items or for global health (Table 4). The largest change was observed in pain scores, which decreased by 8.4 points (27%), and social functioning, which increased by 6 points (10%) at follow-up.

**Figure 3:**
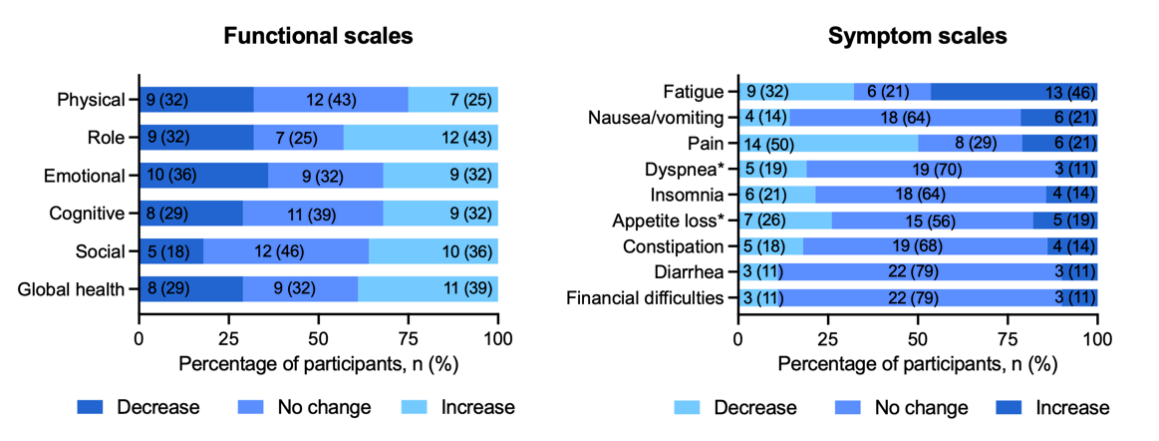
Percentage of individuals with decreased, increased, or unchanged Quality of Life Questionnaire C30 scores on the functional and symptom scales. *1 missing value; n=27 answers were analyzed.

Functioning in all subcategories remained the same or increased in approximately two-thirds of the participants, with most participants seeing an improvement in role functioning (Figure 3). Most participants (15-22 of 28, 56%-79%) saw no change in symptoms, except for pain, which decreased in 50% (12/28) of participants, while fatigue increased in 46% (13/28) of participants by the end of 4 weeks.

**Table 5:** Quality of Life Questionnaire C30 scores for each item scale at inclusion and follow-up.

Domain		Inclusion (n=28)	Follow-up (n=28)	*P* value
**Functional scales, mean (SD)**				
	Physical	85.5 (10.7)	83.8 (14.1)	.39
	Role	60.7 (20.9)	62.5 (26.7)	.54
	Emotional	72.3 (16.5)	73.5 (18.0)	.63
	Cognitive	69.0 (21.6)	70.8 (20.6)	.65
	Social	58.3 (23.4)	64.3 (21.6)	.21
**Symptom scales, mean (SD)**				
	Fatigue	35.7 (14.3)	40.1 (22.1)	.55
	Nausea or vomiting	8.3 (12.4)	8.9 (14.0)	.67
	Pain	31.0 (20.1)	22.6 (23.2)	.16
	Dyspnea^a^	22.2 (22.6)	23.5 (27.4)	.94
	Insomnia	31.0 (25.5)	29.8 (27.7)	.87
	Appetite loss^a^	19.8 (19.1)	19.0 (26.3)	.48
	Constipation	11.9 (22.6)	11.9 (20.7)	.85
	Diarrhea	11.9 (20.7)	10.7 (18.3)	.74
	Financial difficulties	19.0 (30.7)	15.5 (21.2)	.52
Global health status, mean (SD)		61.6 (17.0)	61.9 (15.8)	.78

^a^1 missing value; n=27 answers were analyzed.

#### In-App PROs

**Figure 4:**
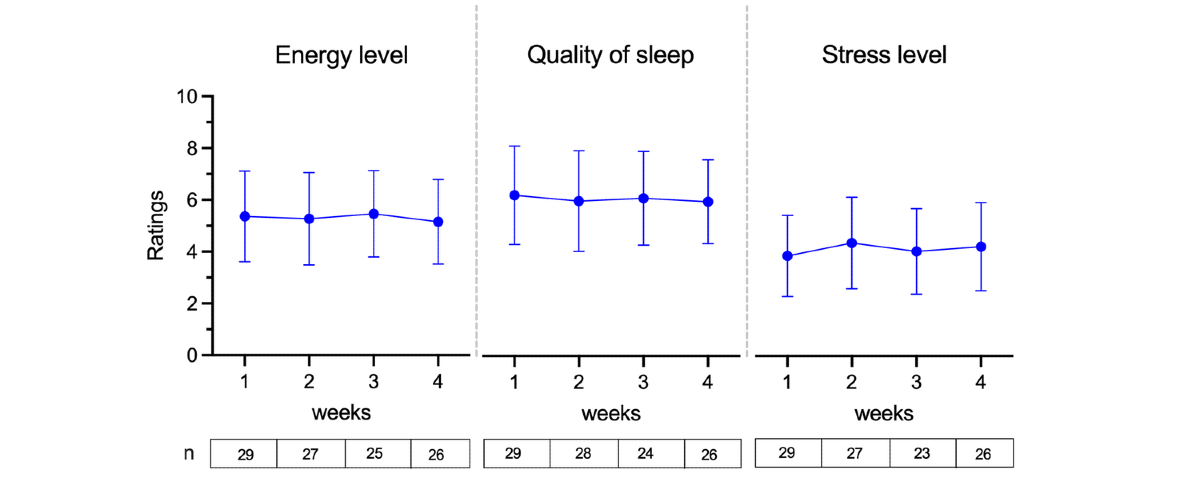
Average weekly changes in users’ ratings of energy levels, quality of sleep, and stress levels (error bars show SD), with the number of users reporting these (n) each week shown under the graphs.

PROs on energy levels, quality of sleep, and stress levels collected within the app showed that they remained stable over time, with no significant changes (Figure 4). In week 1, all 29 users reported that these PROs and engagement remained high, as 26 users still engaged with them in week 4.

### Exploratory Analysis

**Figure 5:**
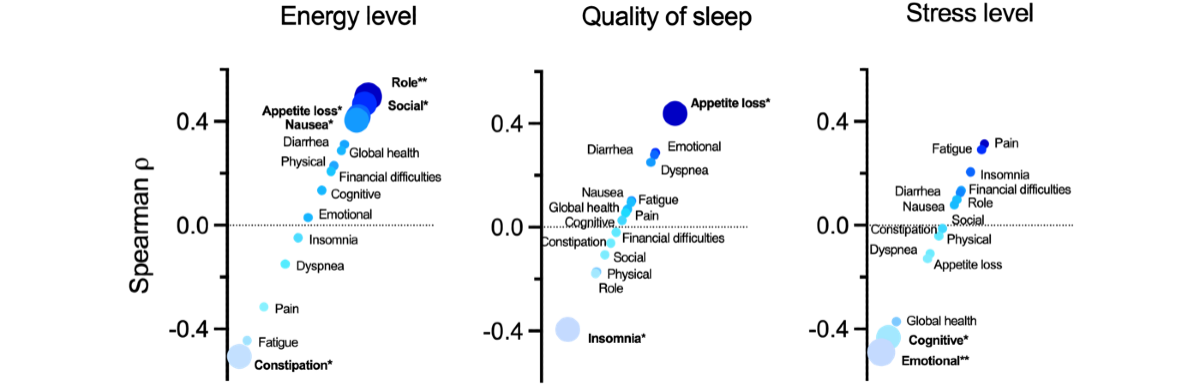
Spearman rank correlation analysis of in-app quality of life (QoL) and Quality of Life Questionnaire C30 (QLQ-C30) item scores. The listed QLQ-C30 items below 0 are inversely correlated, while above 0, they are positively correlated with energy level, quality of sleep, and stress levels. Items that significantly correlate with either in-app QoL measure are shown in bold and are represented by large circles, while those items with no significant correlations are represented by small circles. *P≤.05; **P≤.01.

We carried out an exploratory correlation analysis between the QLQ-C30 and in-app–reported QoL measures to gain insight into the clinical validity of the QoL outcomes measured in the app (Figure 5). We found that energy levels correlated most strongly with role (ρ=0.49; *P*=.007) and social functioning (ρ=0.47; *P*=.01) as well as with nausea, vomiting and appetite loss (ρ=0.41; *P*=.03 each), while it inversely correlated with fatigue (ρ=−0.44; *P*=.02) and constipation (ρ=−0.5; *P*=.006). Quality of sleep significantly correlated with appetite loss (ρ=0.44; *P*=.02) and negatively correlated with insomnia (ρ=−0.39; *P*=.04), while stress levels significantly inversely correlated with cognitive (ρ=−0.43; *P*=.02) and emotional (ρ=−0.49; *P*<.01) functioning.

## Discussion

### Principal Findings

This trial tested the feasibility of a DTx intervention targeted at patients with cancer in active treatment and gathered preliminary information on its effectiveness. We obtained encouraging results regarding retention and engagement; the program had a very high completion rate (97%) and high acceptability (>80%), and thus, the feasibility criteria were met. Engagement metrics painted a similar picture, with users staying active 96% of the time, or 27 out of 28 days, and completing on average 7.7 (SD 1.9) missions a day.

These engagement metrics are somewhat higher than those reported in other studies. A recent systematic review of 6 studies found that the average retention rate of digital behavioral interventions was 90.7% among cancer survivors [28]. However, 1 trial with a patient population similar to ours reported a 50% retention rate during a 6-week intervention and 80% questionnaire completion at follow-up, with 51% to 76% engagement with their app (the amount of content viewed) [21]. Another trial reported that only 41% to 65% of the participants logged in more than twice during the 10-week intervention period when they were not given personalized messaging; however, all those receiving personalized messaging used the app at least twice [29]. Similarly, a 12-week pilot research of breast cancer survivors revealed that 70% of the participants were continuously using the app, with 7.26 log-ins on average in the first month, which later declined [30]. Given that most participants in this study interacted with the app nearly daily, we can conclude that it succeeded in motivating patients with cancer to continually engage with its content. Multimedia content and tailoring, as well as reminders and personalized messaging, were found to increase user engagement with web-based tools [29,31]. These elements were all used by the Sidekick app, which may help explain the relatively low attrition. In addition, the app was designed to associate the completion of missions with appealing sounds or visuals (ie, users receive a supporting animation when they complete a mission) and charitable donations, which in itself might motivate users and increase their engagement. However, cancer survivors are often internally motivated to seek help and use digital interventions to improve their health and fight the disease [32].

In terms of goal attainment, >70% of users were able to achieve their step goals in this trial, and the average step count (approximately 3000-3500 per day) was comparable with that found in other studies with patients in active cancer treatment [9,33-35]. Although these results might underestimate participants’ real step counts (as these had to be manually claimed each day), they suggest that users are interested in tracking their physical activity, particularly their step counts.

The patients in this study were on average more obese than similar patient populations in other trials [9,36,37], and their average BMI was higher than that recommended for cancer survivors [38]. Regarding body composition, normative data are available for healthy adults in Sweden, Switzerland, and wider Europe [39,40]. According to these data, normal body fat percentage falls between 19% and 25% for males and 26% and 36% for females—the results found in our study (26.5% body fat in males and 41% in females) were somewhat higher than these normative values. Regarding cardiorespiratory fitness, reference values for a normal VO_2max_ range (30-43 mL/kg/min for males and 28-34 mL/kg/min for females) have been published for healthy Swedish and Norwegian adults [41,42]. The VO_2max_ values of patients with cancer in this study (28 mL/kg/min for females and 31 mL/kg/min for males) fell on the lower end of these ranges, suggesting lower cardiorespiratory fitness. Although we did not find significant changes in physical fitness or body composition, other studies promoting exercise intervention for patients in active treatment found significant improvements after 6 to 12 weeks [9,43]. Improving physical fitness and reducing sedentary behaviors can improve health-related QoL and reduce the risk of hospital readmissions [8,36,38]; thus, these will be important outcomes in future longer RCTs.

### QoL Outcomes

The global QoL of patients with cancer found in this study agrees with scores reported from patients with cancer in previous studies [9,37,44]. Compared with reference values from a large sample of patients aged 50 to 59 years with breast cancer or cancer in general, our participants scored slightly lower on role, cognitive, and social functioning and had higher symptom burden in most items [45]. An important question, however, is what scores represent clinically significant problems or symptom burden for patients. A previous study sought to answer this question and established cutoff values for 4 subitem scales [46]. According to that study, scores <83 for physical and <70 for emotional functioning and >39 for fatigue and >25 for pain (the most commonly reported symptoms among patients with advanced cancers [47-49]) likely mean significant problems for patients. Compared with these cutoff values, our participants reported less pain by the end of the program, suggesting that pain caused less clinically significant burden to them. In addition, they reported better physical and emotional functioning both before and after the program. Overall, these results showed a generally stable health-related QoL during these 4 weeks, with a trend for improved pain scores. Future RCTs should further evaluate the effectiveness of the program in improving or maintaining QoL. In the long term, even maintaining stable health can be important for patients with cancer, as they usually experience a decline during prolonged treatments or as the disease progresses [50,51].

We found that the in-app PROs positively or negatively correlated with certain QLQ-C30 items as expected. Higher energy levels indicated higher role and social functioning and lower fatigue and constipation, while higher stress levels indicated lower cognitive and emotional functioning. Surprisingly however, higher energy levels were also associated with increased loss of appetite and nausea or vomiting, and better sleep was associated with reduced appetite. It is important to further assess these associations in larger sample trials to better validate PROs used by the Sidekick app.

### Strengths and Limitations

The strengths of this study were excellent retention, engagement, and questionnaire completion, which eliminated the need to correct for missing data. Feedback from the patients suggested that the supportive and familiar environment at the rehabilitation clinic could have played a key role in this finding. An additional strength is the multidisciplinary nature of the intervention, which has been shown to benefit the rehabilitation of patients with breast cancer [37].

A limitation of this study was the small sample size, which was composed of self-selected and likely self-motivated individuals. This restricts the generalizability of the results and reduces the study power for testing preliminary program efficiency. Another limitation arising from the study design was the short program duration of 4 weeks, which is likely too short a time frame to detect significant changes in physical and mental health parameters. Finally, the known limitations of the app are the lack of automatic step counting and the fact that step count missions could not be completed retrospectively for previous days; thus, if users did not claim their steps, the records showed 0 steps for the given day. Therefore, this feature likely underestimated the real physical activity that participants completed and hence should be further optimized in future programs and trials.

### Conclusions

On the basis of evidence gathered, digital support delivered through the Sidekick app is feasible for patients with cancer, and a large-scale RCT can be initiated. Preliminary results suggest that participants’ health-related QoL remained stable for 4 weeks, but a longer, controlled trial will be required to gauge the efficacy of the digital intervention for improving QoL. Changes in the most burdensome side effects, fatigue and pain, should also be the primary focus and assessed using specific measures in future trials. In addition, the digital program could be further tailored to the cancer experience by including education about treatments and specific side effects, providing symptom tracking and medication reminders, and adapting the assigned daily tasks and workload to the individual’s stage on the cancer treatment journey and actual energy and motivation levels.

## Appendix

Multimedia Appendix 1 Details of the weekly education content.

## Abbreviations

DTx: digital therapeutics
PRO: patient-reported outcome
QLQ-C30: Quality of Life Questionnaire C30
QoL: quality of life
RCT: randomized controlled trial
VO_2max_: maximum oxygen uptake
