# Supplementary material for: Evaluating the Feasibility of a Digital Therapeutic Program for Patients With Cancer During Active Treatment: Pre-Post Interventional Study
Source: JMIR Form Res. 2022 Oct 13;6(10):e39764. doi: 10.2196/39764 (PMC9614627; doi:10.2196/39764)
Supplement: Multimedia Appendix 1 [file formative_v6i10e39764_app1.docx]

**Multimedia Appendix 1**

**Evaluating the Feasibility of a Digital Therapeutic Program for Patients with Cancer During Active Treatment: Pre-post Interventional Study**

JMIR Formative Research

**Table S1** Details of the weekly education content.

| **Education topics breakdown** | |
| --- | --- |
| Overarching theme | Daily educational content topics |
| Week 1—mindfulness | Introduction and guide to implementing mindfulness and meditation into the daily routine and importance of self-compassion |
| Week 2—sleep | Importance of getting enough sleep, signs of sleep deprivation, and good practices to improve sleep habits |
| Week 3—stress | The role of acute stress, effects of prolonged stress, how to handle it, and relabeling the stress response |
| Week 4—nutrition | Hydration, portion control, importance of proteins, mindful eating, and building healthy eating habits |
